# Resilience and stability of kelp forests: The importance of patch dynamics and environment-engineer feedbacks

**DOI:** 10.1371/journal.pone.0210220

**Published:** 2019-01-25

**Authors:** Cayne Layton, Victor Shelamoff, Matthew J. Cameron, Masayuki Tatsumi, Jeffrey T. Wright, Craig R. Johnson

**Affiliations:** Institute for Marine and Antarctic Studies, University of Tasmania, Hobart, Australia; University of Sydney, AUSTRALIA

## Abstract

Habitat forming ‘ecosystem engineers’ such as kelp species create complex habitats that support biodiverse and productive communities. Studies of the resilience and stability of ecosystem engineers have typically focussed on the role of external factors such as disturbance. However, their population dynamics are also likely to be influenced by internal processes, such that the environmental modifications caused by engineer species feedback to affect their own demography (e.g. recruitment, survivorship). In numerous regions globally, kelp forests are declining and experiencing reductions in patch size and kelp density. To explore how resilience and stability of kelp habitats is influenced by this habitat degradation, we created an array of patch reefs of various sizes and supporting adult *Ecklonia radiata* kelp transplanted at different densities. This enabled testing of how sub-canopy abiotic conditions change with reductions in patch size and adult kelp density, and how this influenced demographic processes of microscopic and macroscopic juvenile kelp. We found that ecosystem engineering by adult *E*. *radiata* modified the environment to reduce sub-canopy water flow, sedimentation, and irradiance. However, the capacity of adult kelp canopy to engineer abiotic change was dependent on patch size, and to a lesser extent, kelp density. Reductions in patch size and kelp density also impaired the recruitment, growth and survivorship of microscopic and macroscopic juvenile *E*. *radiata*, and even after the provisioning of established juveniles, demographic processes were impaired in the absence of sufficient adult kelp. These results are consistent with the hypothesis that ecosystem engineering by adult *E*. *radiata* facilitates development of juvenile conspecifics. Habitat degradation seems to impair the ability of *E*. *radiata* to engineer abiotic change, causing breakdown of positive intraspecific feedback and collapse of demographic functions, and overall, leading to reductions in ecosystem stability and resilience well before local extirpation.

## Introduction

Coastal marine ecosystems are under threat from a variety of anthropogenic stressors including urbanisation, pollution and climate change [1–3]. One critical effect of these stressors is to reduce the extent and abundance of habitat-forming species, or ‘ecosystem engineers’, such as coral, kelp and seagrass [4, 5]. Ecosystem engineers are of disproportionate importance to the health and function of ecosystems because they create physically complex habitats that support biodiverse and productive communities [6, 7]. In some cases, anthropogenic and environmental stressors can trigger declines in the abundance of ecosystem engineering species to a point where complex and diverse ecosystems ‘phase shift’ to become structurally simple, less diverse and less productive environments [8–11]. It follows that the informed management and conservation of coastal environments requires a thorough understanding of the stability and resilience of marine ecosystem engineers.

Ecosystem engineers modify properties of the local environment via three main pathways: structural engineering (e.g. provision of physical structure); abiotic engineering (changes to the abiotic environment, e.g. light, water flow, nutrients); and biotic engineering (changes in biota as a response to structural or abiotic engineering) [12]. These environmental modifications have major consequences for the ecological community and can alter resource flows [13], ameliorate physical stressors [14], and promote biodiversity [15]. Far less attention however has been given to how engineering of the environment feeds back to influence the engineer itself, i.e. the so-called ‘environment-engineer feedback’ [12].

Environment-engineer feedbacks are predicted to occur when the demography of the engineer is itself affected by the environment it modifies, and intraspecific facilitation arising from these feedbacks is likely to apply to many habitat-forming species that reproduce, recruit, and grow in the engineered habitat [12, 16, 17]. However, these feedbacks are likely to be complex, not only because of the potential for synergistic or interactive effects among the separate mechanisms (i.e. structural, abiotic, biotic) [12], but also because ecosystem engineering (and thus any feedbacks) are likely density and/or patch-size dependent [18–20]. Ecosystem engineering is also likely to be context-dependent and vary across environmental gradients to become more important in extreme environments [12, 14, 21]. While these complexities are recognised at a theoretical level, the net consequences of these feedbacks for species have not yet been explored empirically.

Kelp (Order Laminariales) dominate coastal environments in temperate and subpolar latitudes around the globe [2]. These ecosystem engineers create complex habitats that support diverse and productive communities [22, 23], and modify local abiotic processes such as light, sedimentation and water flow [20, 24, 25]. In numerous regions globally, kelp forests are in decline and experiencing reductions in patch size and kelp density [2, 22, 26]. It is therefore imperative to understand how habitat degradation and anthropogenic stressors influence the resilience and stability of kelp as marine ecosystem engineers.

The most widespread and abundant habitat-forming kelp in Australasia is *Ecklonia radiata* [27]. This stipitate kelp [28] rarely grows taller than 1.5 m, but dominates the Great Southern Reef–Australia’s continental wide temperate reef system–and supports high levels of biodiversity and endemism [29]. Like many kelp globally, *E*. *radiata* is under threat from rising ocean temperatures, overgrazing from invasive and range-expanding species, and urbanisation and pollution [5, 8, 30, 31]. As a result, this species is becoming increasingly sparse and patchy in many locations across its range. Existing research into the resilience of kelp, and most marine ecosystem engineers, has focussed primarily on their response to external processes such as disturbance or interspecific interactions [32–34]. Despite this, it seems that internal drivers influencing kelp demography–and particularly positive environment-engineer feedbacks–may be equally important. Indeed, a breakdown of positive environment-engineer feedbacks caused by reductions in patch size or adult density may explain the slow recovery of kelp often observed after large-scale losses [30, 33, 35]. Further, there are a number of critical gaps in current understanding of kelp demography, for example there have been few demographic studies of the microscopic life stages of *E*. *radiata*, particularly involving recruitment and survival of microscopic sporophytes *in situ* [36].

This study determined how ecosystem engineering by the kelp *E*. *radiata* is affected by reductions in patch size and kelp density, and whether this feedbacks to influence the species’ demographic rates. We constructed an array of artificial reefs of different sizes onto which adult kelp were transplanted at a range of densities. We then examined (i) how the physical environment beneath *E*. *radiata* canopies changed with reductions in patch size and adult kelp density and, (ii) how these affected fundamental demographic processes of microscopic and macroscopic juvenile *E*. *radiata* including recruitment, survivorship and growth.

## Materials and methods

### Field site and artificial reefs

Research was completed under permits #14130, #16202 and #H303729, issued by Tasmanian Department of Primary Industries, Parks, Water and Environment. The experimental site (-42.64693, 148.01481) was a semi-exposed, sandy embayment off Maria Island on the east coast of Tasmania, Australia. This area was selected for its uniform depth (6.5 m) and isolation from natural rocky reefs (>1.5 km). Here, divers using SCUBA installed artificial reefs to provide structure and substratum for kelp transplanting. Artificial reefs were constructed in seven patch sizes (0.12, 0.24, 0.48, 1.08, 1.92, 4.32 and 7.68 m^2^) and crossed with four kelp density treatments (0–17 kelp/m^2^, details below) to produce 28 distinct experimental patch reefs with a total combined area of ~63 m^2^. *Ecklonia radiata* habitats often contain patches of this size, especially following habitat degradation (e.g. urchin overgrazing) where the canopy is disturbed and fragmented prior to extirpation [31, 37, 38]. All patches had a length-width ratio of ~4:3 and consisted of a steel frame and concrete Turfstone pavers (400 x 300 x 100 mm, L x W x H). The reef frames were elevated 30 cm above the substratum on legs to eliminate sand inundation and allow drift algae to pass underneath.

**Fig 1:**
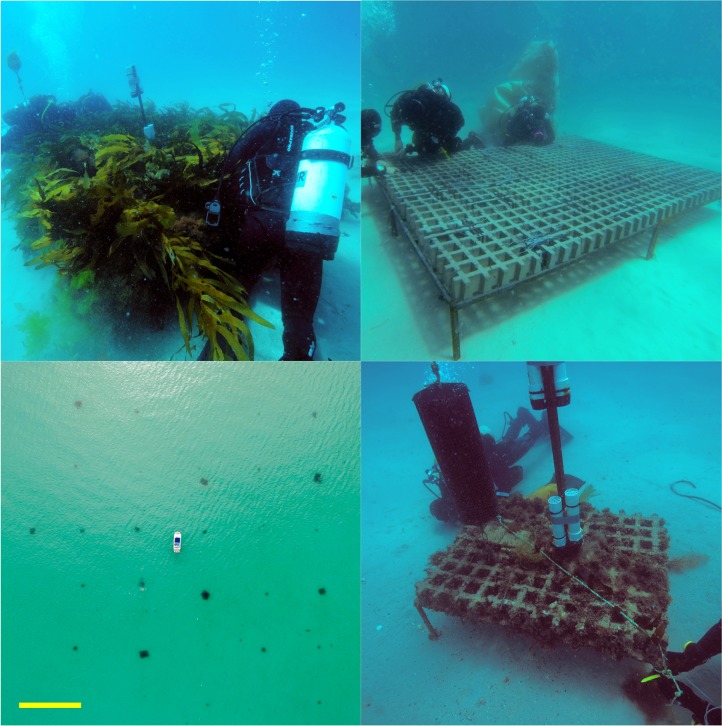
Experimental patch reef array. Clockwise from top left: a 7.68 m^2^ patch reef with transplanted kelp; a 7.68 m^2^ patch reef during construction; a 1.08 m^2^ patch reef with no transplanted kelp, and; an aerial photo of the grid formation of experimental patch reefs (yellow scale bar represents 25 m).

Reefs were installed in December 2014 in a grid consisting of 5 columns and 6 rows and spanning >12,500 m^2^ (Fig 1). Each reef was randomly allocated a position within the grid and separated from neighbouring reefs by 25 m. Effective dispersal distances in kelp are typically proposed to be short and occur over metres [28, 39, 40], and although longer distance dispersal can occur under certain conditions [40], molecular data suggests low dispersal in *E*. *radiata* [41]. As such, each reef was considered isolated from receiving meaningful amounts of spores from neighbouring reefs (see ‘Results‘ for confirmation of this assumption).

### Kelp transplanting

Adult *E*. *radiata* were transplanted to the reefs at four different densities, based on a mean density of adult kelp on nearby natural reefs of ~8 /m^2^. The treatment levels were no kelp, half-natural, natural, and double-natural kelp density. Due to the constrained dimensions of the pavers, the specific kelp densities were 0, 4.1, 8.3 and 16.6 kelp/m^2^, which are hereafter called zero, low, medium and high density, respectively. Stands of *E*. *radiata* naturally contain areas with this range of adult kelp densities [20, 35, 42].

Adult sporophytes were collected at depths of 4–8 m from the closest extensive population of *E*. *radiata* (~9 km from the experimental site) and transplanted onto the reefs during January and February 2015. All transplanted *E*. *radiata* were stage 3 adult sporophytes (*sensu* [35]) with a stipe between 150–300 mm in length, and with no substantial epibiotic growth or degraded tissue. Sporophytes were collected by removing their holdfast from the substratum using a blunt-tip knife and placed in large opaque bags before being transported to the experimental site and transplanted, generally within 6 hours (always <18 hrs).

The holdfasts of the transplanted kelp were secured to the reefs using large rubber bands, and after 4–6 weeks, newly developed haptera had reattached the holdfast to the concrete pavers. In total, 462 adult sporophytes were initially transplanted to the experimental site. Kelp density treatments were maintained every 6 weeks and any lost adult sporophytes were replaced using the same methods as above.

### Abiotic measurements

#### Water flow

The dissolution of plaster clod cards was used to determine relative differences in sub-canopy mass transfer. For the sake of communication, mass transfer is hereafter referred to as water flow, but we acknowledge the difference of these processes [43, 44]. Clod cards were made by mixing gypsum plaster and fresh water at the manufacturer’s ratio of 10:7.5 plaster-water and cast using hemispherical silicon moulds (~60 mm x 40 mm). Clod cards were left to set for 24 hours and then dried at 50°C for a further 24 hours, before being labelled and weighed.

Each clod card was mounted to a thin plastic base and installed in the centre of the patch, attached either to the substratum or to a bracket atop the sediment trap array. These positions represented sub-canopy and above-canopy environments, respectively. Clod cards positioned above the canopy acted as controls that measured ambient water flow, while those below the canopy measured treatment effects. All clod cards were fitted with a coarse mesh guard to eliminate confounding effects of erosion due to kelp scour. Clod cards were collected after ~72 hours *in situ*, dried at 50° C for 24 hours and then re-weighed. Due to inconsistencies in the plaster, the initial masses of clod cards varied by ~15% and so mass loss was standardised as a percentage of mass lost. Preliminary testing revealed no relationship between initial mass and magnitude of mass lost (Table A in S1 File).

Clod cards were installed during winter (June) and spring (September). These are seasons of maximum water motion in Tasmania due to storms and winds, and peak periods for *E*. *radiata* spore release and recruitment of juvenile sporophytes [34, 45, 46].

#### Irradiance

Photosynthetically Active Radiation (hereafter, irradiance) was measured using a LI-COR LI-1500 Light Sensor Logger and a LI-193 Spherical Underwater Sensor. This 330° sensor allowed measurements of the incidental irradiance that entered through the sides of the kelp patches. The sensor was secured to a 1.8 m pole to minimise diver interference, and irradiance was recorded for 60 seconds above and below the kelp canopy in the centre of each patch. Irradiance was automatically logged every 15 seconds as mean μmol photon m^-2^ s^-1^, providing four mean measures per position over the 60-second sample. Measurements above the kelp canopy recorded ambient irradiance and acted as controls, while sub-canopy readings measured treatment effects. Measurements were taken on a fine day in spring (November) between 1030–1430 hours. Preliminary analyses confirmed no effect of time of day on measurements (Table A in S1 File).

#### Sediment deposition

Sediment deposition on each patch was measured using sediment traps constructed from PVC piping. Each trap was cylindrical (300 x 50 mm, L x D) and had a baffle installed across the opening. Sediment traps were installed in autumn (April), winter (July), spring (September) and summer (December) for a period of 41 days (± 2, SE). Four traps were positioned in the approximate centre of each patch, with two traps above the kelp canopy and two below. Above-canopy traps acted as controls that measured background rates of sediment deposition, while sub-canopy traps measured treatment effects.

For collection, sediment traps were sealed underwater before being retrieved and transported to the lab, where trap contents were flushed into pre-weighed foil trays and dried at 70°C until a constant mass (~48 hours). The dried sediments were weighed on a laboratory balance (to 0.01 g) and a rate of deposition above and below the kelp canopy in each patch was calculated based on the mean dry mass of the sediment from the two traps in each position (g day^-1^ m^-2^). When a trap was lost or unsuitable for analysis (e.g. due to octopus habitation), the sediment mass was calculated from a single trap. Dried sediments were sieved through 250 and 62 μm mesh to examine their composition.

#### Sediment accumulation

The depth of accumulated sediments covering the substratum was measured to the nearest 1 mm using a small ruler at five locations in each patch–once in the approximate centre of the patch and four more at randomly selected compass marks approximately halfway to the patch edge. Sampling occurred in spring (September) and summer (January).

### Kelp demographics

#### Macroscopic juvenile sporophytes

Macroscopic juvenile *E*. *radiata* sporophytes were transplanted onto a subset of patches, and their survivorship and growth determined over a 3-month period. A subset of patches was used due to limitations in the number of juveniles that could be collected. In autumn (April), juvenile sporophytes were transplanted across all patch sizes from the medium density treatment. To determine kelp density effects, the subset of patches for the following seasons (winter, spring and summer) included all kelp density treatments but fewer patch sizes (Table B in S1 File). Storms prevented collection of sufficient data during winter, so no data were analysed from that season.

For transplanting, stage 1 juvenile sporophytes 50–150 mm in length were collected from the same site as the adult kelp. Each juvenile was measured to the nearest 2 mm and hole-punched at the base of the lamina above the meristem to determine growth. Ten sporophytes were installed in each selected patch by threading their holdfasts into the twine of nylon rope at 100 mm intervals, and the rope secured in the patch centre. After 40 ± 3 days (SE), growth of each transplanted juvenile was measured to the nearest 2 mm *in situ*, from the top of the holdfast to the base of the punched-hole, and the sporophyte hole-punched again at the original position. If a sporophyte was missing, that individual was recorded as having not survived. After 90 ± 9 days (SE) the ropes were collected, and growth and survivorship determined again. Growth rates over the entire transplant period were calculated by summing the first and second growth measurements and expressed as mm/week. Preliminary analyses revealed growth rates of juvenile sporophytes were linear and independent of initial length (Table A in S1 File).

#### Microscopic sporophyte recruitment and survivorship

Like all kelp, mature *E*. *radiata* release motile zoospores that settle and germinate into male or female gametophytes [40, 47]. Once fertilised, female gametophytes provide the base from which the microscopic sporophyte develops.

Lab-cultured microscopic sporophytes were outplanted during peak recruitment in winter (June) to determine survivorship across patch size and adult density. Blank microscope slides deployed at the same time measured natural background recruitment of microscopic *E*. *radiata* sporophytes.

Reproductive tissue for culturing was collected from stage 3 *E*. *radiata* at the collection site, and cultured following previous methods [36, 45]. Briefly, zoospores at a density of ~7000/mL were settled onto fully-frosted microscope slides submerged in UV-sterilized and filtered (0.2 μm pore-size) seawater, and the culture maintained for 43 days. Following this period, 10 randomly selected slides were assessed under microscope to determine mean sporophyte abundance prior to outplanting, which was ~7700 (± 550, SE) sporophytes per slide. Six randomly selected slides with cultured sporophytes, and two blank fully-frosted microscope slides (which had been curing in filtered seawater during the culturing process and acted as controls that measured natural recruitment) were then attached in random order to a plastic rack. Racks were transported submerged in seawater in an insulated container to the experimental site where divers attached two racks (i.e. 12 slides supporting sporophytes and 4 control slides) in the approximate centre of each patch. After 42 days, racks were collected and transported to the lab and the number of sporophytes on each slide counted. The duration of the outplanting was sufficient for naturally settled zoospores to have developed into microscopic sporophytes [40, 45].

#### Recruitment of macroscopic sporophytes

Natural recruitment of macroscopic juvenile sporophytes onto each patch was determined via visual census. During a timed search (5 seconds per paver) all visible (approximately >5 mm) stage 1 *E*. *radiata* sporophytes were counted on each patch. Censuses were conducted by the same individual (CL) and occurred during peak recruitment in spring (November), the following winter (June), and at the end of the study in spring (November) 2016. Only the data from the end-of-study census was analysed, as this was the time of highest overall recruitment–nonetheless these data mirrored the patterns from the earlier censuses (Fig I in S1 File).

### Data analysis and statistics

Because the first aim was to test the ecosystem engineering capacity of *E*. *radiata*, we analysed levels of sub-canopy light, sediment deposition and water flow *relative* to the above-canopy environment. Preliminary tests confirmed no effects of experimental treatments on above-canopy abiotic conditions (Table C in S1 File). To simplify analyses and temporal-autocorrelation, abiotic data were time-averaged across seasons (other than irradiance data that were only collected in spring), where replicates represent the number of seasons when measurements were taken (water flow *n* = 2; sediment deposition *n* = 4, and; sediment accumulation *n* = 2). In each case, time-averaged data reflect the same patterns observed in seasonal data (see Figs E–H & Table D in S1 File).

Because macroscopic juvenile *E*. *radiata* were not transplanted into all patches each season (see Table B in S1 File), and low/no survivorship in some treatments (see Results), it was not possible to analyse all planned treatment combinations. Consequently, using the limited dataset we analysed (i) whether survivorship differed across all patch sizes of the medium kelp density treatment; (ii) whether survivorship differed across all kelp density treatments using the subset of patch sizes from spring and summer, and (iii) whether survivorship differed across seasons using the subset of patches of medium density. Analysis of growth rates of transplanted macroscopic juveniles necessitated a similar approach, and we tested: (i) whether growth rates differed across all seasons using the subset of patches of medium kelp density, and (ii) whether growth rates differed among the kelp density treatments using the subset of patch sizes from spring only

Data were primarily analysed using Analysis of Covariance (ANCOVA) with patch size as a fixed covariate. Model factors were fixed, and Type III (i.e. partial) Sums of Squares were used since they are appropriate for both balanced and unbalanced data, as occurred in our datasets. Tests assumptions were assessed using diagnostic plots of model residuals and data were transformed when necessary based on values of λ from Box-Cox plots, which are noted in model output. Similarly, the covariate (patch size) was log_2_ transformed when it improved conformity to test assumptions, reflecting that the span of patch sizes followed a log_2_ scale. The standard process for ANCOVA was followed whereby the saturated model including the interaction term was first tested for homogeneity of slopes, before the unsaturated model without the interaction term was fitted when homogeneity of slopes was upheld. If the saturated model did not show homogeneity of slopes and it was statistically and biologically appropriate, the least homogenous treatment was omitted and the model re-run using the same approach. Regression slope analysis was also used and is noted in model output. Analyses were conducted using *MASS* and *car* packages in *R* (v. 3.2.5; *R* Core Team), with alpha at α = 0.05. Figures were produced using *ggplot2* in *R*, and *Inkscape* (v. 0.91), and for clarity are presented using untransformed response variables and without 95% confidence intervals.

## Results and discussion

### The abiotic environment

#### Water flow

**Fig 2:**
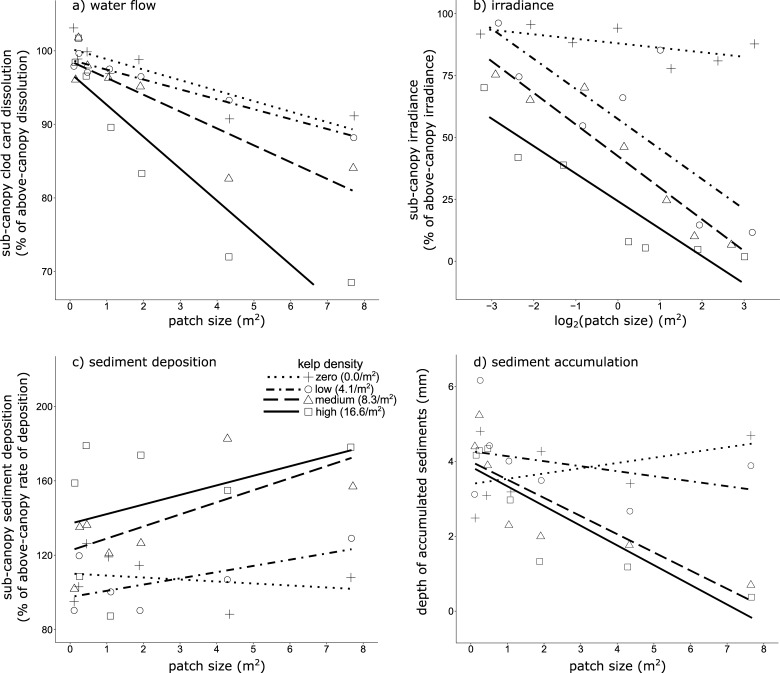
Effects of *Ecklonia radiata* patch size and kelp density on sub-canopy abiotic factors. (a) Water flow (expressed as dissolution of plaster clod-cards relative to the paired above-canopy clod-card); (b) irradiance levels (expressed as light relative to the paired above-canopy measurement, where mean ambient irradiance was 558 ± 26 μmol photon m^-2^ sec^-1^ , *n* = 112, ± SE); (c) sediment deposition (expressed as percentage of sediment deposition relative to the deposition recorded in a paired sediment trap above the canopy), and; (d) depth of accumulated sediments in turf-sediment matrices growing on the reef substratum (mm).

The interaction between patch size and density of *Ecklonia radiata* in influencing sub-canopy clod card dissolution was significant (ANCOVA; *F*_(1,24)_ = 13.605, *P* = 0.001); reflecting that while sub-canopy dissolution (and thus water flow) decreased with patch size, the magnitude of the reduction increased with kelp density (Fig 2A). The high density treatment showed the most marked reductions in sub-canopy flow with increasing patch size, and in the largest patch from this treatment, sub-canopy flows were ~70% of those external the canopy. This is somewhat less than the only other published measure of flow beneath a stipitate kelp canopy of which we are aware [25]–which used plaster clod cards to illustrate that beneath a kelp canopy of *Agarum* spp. and *Saccharina latissima* at 7–11 m depth, sub-canopy flows were ~50% of ambient conditions. Nonetheless, our experimental kelp patches were smaller than the natural habitats investigated by that previous work, and so the capacity of natural stands of *E*. *radiata* to reduce environmental water flow is likely greater than our results indicate. Indeed, sub-canopy flows within deeper (~14 m depth) and larger (>70 m^2^) patches of *E*. *radiata* in Tasmania can be 40–60% of ambient conditions [Layton et al. in prep.].

Omitting the high kelp density treatment from the model eliminated the significant interaction, and the unsaturated model revealed significant main effects of patch size and kelp density on sub-canopy water flow (Fig 2A, Table 1). The strong effect of patch size in modifying sub-canopy flow suggests that water entering through the sides of *E*. *radiata* patches is a strong determinant of sub-canopy hydrodynamics. Previous work [25] demonstrates that tracer particle penetration through the kelp canopy (and thus mass transfer and water flow) is minimal, and supports this interpretation. Baffling of flow by kelp stipes, along with friction on the substratum and underside of the kelp canopy [48, 49] are therefore likely to be key mechanisms in reducing sub-canopy flow.

**Table 1:** ANCOVA tests of effects of kelp density and patch size on abiotic factors below the *Ecklonia radiata* canopy.

*Data analysed*	*factor*	*F (df)*	*P*
**WATER FLOW** w/o high density (*Y*)^2.50^	kelp density	F_(1,18)_ = 4.628	0.045*
	log_2_(patch size)	F_(1,18)_ = 45.259	<0.001*
**IRRADIANCE** (*Y*)^0.15^	kelp density x log_2_(patch size)	F_(1,108)_ = 71.120	<0.001*
**SEDIMENT DEPOSITION** w/o zero density (*Y*)^0.75^	kelp density	F_(1,18)_ = 9.825	0.006*
	patch size	F_(1,18)_ = 5.941	0.025*
**SEDIMENT ACCUMULATION** (*Y*)^0.50^	kelp density x log_2_(patch size)	F_(1,24)_ = 22.908	<0.001*

Output is for either saturated models (where homogeneity of slopes was rejected) or unsaturated models after confirming homogeneity of slopes. Response variable (*Y*) and associated transformation noted in the first column.

Significant *P*-values denoted in bold and with *.

Increasing densities of kelp also significantly reduced sub-canopy flow (Table 1). Given that the capacity of the different kelp densities to modify flow was most pronounced in the largest patches, kelp density may be a more important factor in flow-modification in patches beyond the size of our experimental reefs. Kelp density effects may also be influenced by seasonal differences in canopy biomass–which for *E*. *radiata* is typically highest in spring and early summer and lowest in autumn and early winter [50, 51]. Indeed, seasonal analyses reflected this pattern, with stronger effects of kelp density on sub-canopy flows in spring rather than winter (Table D in S1 File).

Regression slope analysis of the zero density treatment illustrated that clod cards positioned in the sub-canopy position on reefs without kelp dissolved more slowly with increasing patch size (*F*_(1,5)_ = 16.02, *P* = 0.010). Thus, it appears that the larger reef structures themselves influenced water flow close to their surface, possibly due to eddy formation [44]. Nonetheless, any artefact from the reef structures is unlikely to have influenced the broader results, given the reductions were slight relative to those observed on reefs with adult kelp (Fig 2A).

Ultimately, the canopy of *E*. *radiata* strongly influenced sub-canopy water flow, however the effect diminished as patch size decreased, and patches <1 m^2^ (and regardless of kelp density) showed limited capacity to modify flow (Fig 2A). Degradation of *E*. *radiata* habitats through reductions in patch size and kelp density therefore seems likely to increase sub-canopy water flow in these environments. This has the potential to influence a suite of physiological dynamics [52], and abiotic factors including kelp scour [53, 54] and sedimentation [25, 55] (discussed below).

#### Irradiance

Mean above-canopy irradiance during the sampling period was 558 ± 26 μmol photon m^-2^ sec^-1^ (± SE, *n* = 168) with a maximum of 1417 μmol photon m^-2^ sec^-1^. Relative sub-canopy irradiance declined with patch size, but only in patches supporting kelp (Fig 2B), yielding a significant interaction between patch size and kelp density (Table 1). Removing the zero density treatment–for which there was understandably no relationship between ‘sub-canopy’ irradiance and patch size (*F*_(1,26)_ = 0.444, *P* = 0.511)–did not remove the significant interaction, and so the saturated model was retained. This significant interaction possibly reflected that as patch size and kelp density simultaneously increased the nature of the reduction in sub-canopy light changed from linear decay at smaller patch sizes to negative exponential decay at larger patch sizes (Fig F in S1 File).

Accordingly, the greatest reduction in irradiance occurred in the largest patch supporting the highest density of kelp (Fig 2B), where only 1.8 ± 0.3% (± SE, *n* = 6) of above-canopy irradiance, or 6.2 ± 1.3 μmol photon m^-2^ sec^-1^ (± SE, *n* = 6), reached the sub-canopy. This is similar to previously reported measurements of irradiance beneath *E*. *radiata* canopies at similar depths (7–10 μmol photon m^-2^ sec^-1^; [20, 54])–although these studies used flat PAR sensors.

The exponential reductions to sub-canopy irradiance we observed largely seems due to the importance of patch size in minimising the incidental irradiance that enters through the sides of patches. For instance, despite a sparse kelp canopy, the largest patch supporting kelp at low density had a relatively dark sub-canopy (~15% of above-canopy levels, Fig 2A). In contrast, within the smaller patches supporting kelp at medium or high densities, 50–75% of above-canopy light penetrated to the sub-canopy (279–419 μmol photon m^-2^ sec^-1^_,_ Fig 2A). Irradiance entering from the sides of the patches must therefore comprise a substantial portion of the light that reaches the sub-canopy environment. Thus, previous measurements of light using flat sensors may have underestimated sub-canopy irradiance, especially in small patches of kelp.

The light regime in kelp forests strongly influences the associated community [20, 42], the kelp itself [56] and indirectly, other abiotic factors such as sedimentation [57]. The ability of *E*. *radiata* and other stipitate kelp to modify sub-canopy irradiance is well-recognised [20, 28, 40, 54]. However, we demonstrate that this capacity to regulate sub-canopy irradiance is dramatically reduced as both patch size and kelp density decrease (Fig 2B).

The pattern of our results suggests that large and/or dense areas of *E*. *radiata* may possess a capacity to buffer sub-canopy light in spite of some level of habitat degradation (Fig 2B). We emphasise however that in habitats that have already experienced habitat degradation, further reductions to patch size or kelp density (even when small) can result in dramatic increases in sub-canopy irradiance. This will likely have significant implications for the understorey community and the juvenile life-stages of *E*. *radiata* that develop beneath the adult canopy (discussed below).

#### Sediment deposition

The time-averaged rate of above-canopy (i.e. ambient) sediment deposition was 104.7 ± 7.9 g m^-2^ day^-1^ (± SE, *n* = 108), but varied markedly across seasons (~50–200 g m^-2^ day^-1^). These are within the range of rates of ambient sediment deposition observed on *E*. *radiata-*dominated reefs in southern Australia ([58, 59] Layton et al. in prep.) and rocky reefs elsewhere globally [1]. Sediment deposition however was typically higher below the kelp canopy than above, with relative values >100% (Fig 2C).

Relative sub-canopy sediment deposition increased significantly with kelp density (ANCOVA, *F*_(1,25)_ = 13.422, *P* = 0.001), while patch size had no significant effect, albeit very marginally, on sub-canopy rates of deposition (ANCOVA, *F*_(1,25)_ = 4.193, *P* = 0.051). After removing the zero density treatment–where there was no relationship between sub-canopy sediment deposition and patch size (*F*_(1,5)_ = 0.261, *P* = 0.631)–and examining only the treatments where kelp was present, increasing kelp density and patch size were both found to significantly increase sub-canopy sediment deposition (Table 1, Fig 2C).

Sediment deposition within a kelp forest is a function of the supply of sediment particles available for deposition, water flow, settling velocity of particles, and the ability of the kelp canopy to intercept particles [25, 55, 60]. The source of sediment particles deposited on the experimental patches was likely local resuspended benthic sediments [60], since there are no substantial riverine inputs in the area. Indeed, deposited sediments were mostly fine to very fine sand (250–62 μm). Fine-scale hydrodynamic processes–especially the reduction of sub-canopy flow–were then likely responsible for the particles settling in the sub-canopy [48, 61]. Our data of sub-canopy flow supports this explanation, and the highest rates of deposition typically occurred in the patches with lowest sub-canopy flow (Fig 2).

We conclude that degradation of *E*. *radiata* stands may reduce sub-canopy sediment deposition; however, the magnitude of the impact is likely to be highly dependent on local environmental dynamics. Nonetheless in natural and intact *E*. *radiata* stands, the benthos is already characterised by a distinct absence of sediments [1, 34, 62], and this is because sedimentation is a two-part process involving not only deposition, but also accumulation.

#### Sediment accumulation

We did not observe unbound sediments on the experimental reefs, and sediment particles (which appeared to be of similar composition to those collected in the sediment traps, being mostly fine sands) were always accumulated within algal turfs of red or green filamentous or foliose algae. This turf algae-sediment matrix is typical of sedimentation on rocky reefs in Australia [57, 63] and elsewhere around the world, and the negative effects of sedimentation on reef ecosystems worldwide are well documented [1, 64].

The depth of accumulated sediments on the reefs ranged from 0–11 mm, similar to depths of turf-sediment matrices observed on *E*. *radiata*-dominated reefs in southern Australia ([59] Layton et al. in prep.) and rocky reefs elsewhere globally [65] (0–50 mm). Sediment accumulation on the experimental reefs was influenced by a significant interaction between patch size and kelp density (Table 1). This was because patch size either had no relationship (zero density, *F*_(1,5)_ = 1.058, *P* = 0.351; low density, *F*_(1,5)_ = 0.858, *P* = 0.397) or a negative relationship (medium and high density) with sediment accumulation (Fig 2D), with larger patches having very low amounts of accumulated sediments. No appropriate term could be omitted from the saturated model to remove the significant interaction, and so the original model was retained.

Differences amongst kelp density treatments were likely influenced by interactions with other abiotic factors including light and scour–because these factors influence the formation of the sediment-capturing turf algae [54, 55]. Scour not only suppresses development of algal turfs [55, 66, 67] to indirectly limit sediment accumulation, but also physically removes unbound sediments via the sweeping of the kelp lamina [54]. Medium and high kelp density treatments had an almost exponential increase in sediment accumulation with reductions in patch size, which presents an interesting synergy with the exponential increases of light in degraded kelp habitats (Fig 2). Such non-linear ‘tipping-point’ responses are of particular interest given that productive kelp ecosystems are known to rapidly phase-shift to denuded turf algae-sediment habitats after experiencing degradation [1, 30, 64].

The results complement existing work [20, 54, 55] and illustrate that ecosystem engineering by *E*. *radiata* strongly modifies sub-canopy sediments, with the depth of sediments on the experimental reefs approaching zero with increasing patch size and kelp density (Fig 2D). Interactions between sediment deposition, irradiance, scour, and turf algae (see also 20, 61) seem to have resulted in the paradoxical situation where the least sediment accumulation occurred on those reefs with the highest rates of sediment deposition (Fig 2). This ‘sediment paradox’ suggests that sedimentation within *E*. *radiata-*dominated ecosystems is driven primarily by factors that regulate the capture and accumulation of sediments rather than their supply and deposition. Accordingly, it seems that sedimentation is likely to increase as patch size and kelp density decrease in degraded stands of *E*. *radiata* stands, which is concerning given the negative effects that sediments have on the colonising life stages of *E*. *radiata* and other kelp species [1, 57, 68].

### Kelp demographics

#### Survivorship of transplanted macroscopic juvenile sporophytes

**Fig 3:**
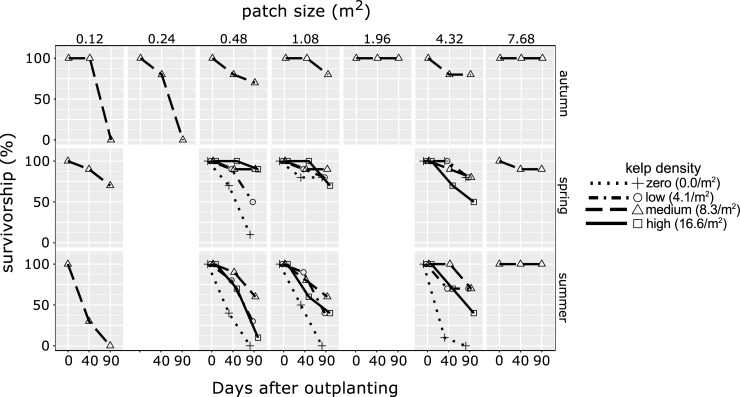
Differences in survivorship of transplanted juvenile *Ecklonia radiata* sporophytes with patch size, season, and kelp density.

During autumn, survivorship of juveniles transplanted to all seven patches of medium kelp density increased significantly with increasing patch size (Table 2, Fig 3). There were no surviving juveniles on the two smallest patches (<0.25 m^2^) after the 90-day period, but survivorship stabilised on patches >1 m^2^ and was always ≥70% (Fig 3).

**Table 2:** ANCOVA tests (and one linear regression^1^) of effects of kelp density, patch size, and season on juvenile *Ecklonia radiata*.

*Data analysed*	*factor*	*SS (df)*	*F*	*P*
**SURVIVORSHIP OF TRANSPLANTED JUVENILE SPOROPHYTES**				
autumn^1^ (*Y+*0.1)^0.50^	log_2_(patch size)	82.68 (1,4)	12.306	0.017*
spring + summer (*Y+*0.1)^0.70^	season	293.85 (1,23)	7.227	0.013*
	kelp density	17.89 (1,23)	0.440	0.514
	log_2_(patch size)	218.09 (1,23)	5.364	0.030*
medium density (spring omitted) (*Y+*0.1)^0.70^	season	3.08 (1,9)	0.101	0.758
	log_2_(patch size)	814.77 (1,9)	26.589	<0.001*
**GROWTH OF TRANSPLANTED JUVENILE SPOROPHYTES**				
medium density (*Y*)^0.60^	season	56.68 (2,110)	41.568	<0.001*
	patch size	9.53 (1,110)	13.977	<0.001*
Spring (*Y*)^0.40^	kelp density	0.07 (1,89)	0.706	0.403
	patch size	0.64 (1,88)	6.719	0.011*
spring + summer (*Y*)^0.55^	season	3.05 (1,131)	6.933	0.009*
	kelp density	1.60 (1,131)	3.641	0.059
	patch size	5.86 (1,131)	13.334	<0.001*
**MICROSCOPIC SPOROPHYTES**				
recruitment (zero density omitted) (*Y+*0.01)^-0.65^	kelp density	98.35 (1,18)	2.729	0.116
	log_2_(patch size)	689.26 (1,18)	19.123	<0.001*
survivorship (zero density omitted) (*Y+*0.01)^-0.15^	kelp density	0.06 (1,18)	1.084	0.312
	log_2_(patch size)	4.31 (1,18)	77.546	<0.001*
**RECRUITMENT OF MACROSCOPIC JUVENILE SPOROPHYTES**				
spring (*Y*)^-0.45^	kelp density	1.40 (1,11)	3.121	0.105
	log_2_(patch size)	6.03 (1,11)	13.440	0.004*
winter (*Y*)^-0.40^	kelp density	2.44 (1,11)	7.214	0.021*
	log_2_(patch size)	5.33 (1,11)	15.766	0.002*
end of study (November 2016) (*Y*)^-0.2^	kelp density	0.19 (1,18)	3.999	0.061
	log_2_(patch size)	2.51 (1,18)	52.693	<0.001*

ANCOVA output is for unsaturated models after confirming homogeneity of slopes. Response variable (*Y*) and associated transformation noted in the first column.

Significant *P*-values denoted in bold and with *.

During spring and summer–when juveniles were transplanted across all kelp density treatments but only a subset of patch sizes–survivorship increased significantly with increasing patch size and was higher in spring than in summer (Table 2, Fig 3). Kelp density did not have a significant effect on survival of juveniles (Table 2), although those transplanted into patches of zero density typically had the highest mortality: especially in summer when none survived on any patches without adult kelp (Fig 3). Juvenile sporophytes transplanted to patches of medium density tended to have the highest survivorship, while those on low and high density patches showed intermediate levels of survival. Seasonal differences in survivorship may be partly explained by seasonal variations in canopy biomass and sub-canopy irradiance; however, this cannot explain why survivorship was also higher on reefs with no adult kelp during spring.

For juveniles transplanted into patches of medium density, there was a significant interaction between season and patch size (ANCOVA; *F*_(2,11)_ = 4.749, *P* = 0.033). Omitting the data from spring–when juveniles displayed the highest survivorship (Fig 3)–eliminated the significant interaction and revealed again that survivorship increased with patch size, but did not differ between autumn and summer (Table 2).

Juvenile *E*. *radiata* are adapted to the low-light conditions typically present beneath intact canopies of adult conspecifics [35, 56, 69]. In fact, small juvenile *E*. *radiata–*along with some species of red algae–are often the only macroalgae that occur beneath dense adult canopies ([20], pers. obs.). Thus, the patterns of survivorship we observed may largely be attributable to irradiance, which we demonstrated is strongly influenced by patch size and kelp density (Fig 2, Table 1).

Canopy removal experiments have shown that juvenile *E*. *radiata* exposed to elevated irradiances (e.g. ~720 μmol photon m^-2^ sec^-1^ *sensu* [56, 69]) experience pigment loss, tissue necrosis and increased mortality. Certainly, those authors’ descriptions of juvenile *E*. *radiata* with bleached holdfasts and eroded lamina or stipes, mirror our own observations. Previous work also demonstrated that high-light stress suffered by juvenile kelp increased as adult density decreased [56]. Of note, is that relative to our own transplanted juveniles, juvenile kelp that recruit and develop within brighter sub-canopies may be more tolerant of increased irradiance due to photoacclimation and/or phenotypic plasticity [62, 69]. No less, we suggest that exposure to increased sub-canopy irradiance is likely to have contributed to the poor survivorship of the juvenile sporophytes on the smaller reefs, and to a lesser extent on the zero and low density treatments.

Elevated irradiance cannot however explain the trend of intermediate survival that we observed on patches of high kelp density (Fig 3). In those instances, survivorship may instead be shaped by inadequate light. Our minimum readings of sub-canopy irradiance, recorded within the two largest reefs from the high density treatment, were very close to the point of photosynthetic compensation for kelp [70]. As such, the quantity of light beneath canopies of high kelp density may have at times been insufficient for photosynthesis by the juvenile sporophytes.

Other abiotic factors might amplify the effects of deleterious irradiance. For example, elevated water flow can cause erosion of *E*. *radiata* sporophytes [25], and may exacerbate the tissue necrosis caused by high light stress. Our observations of increased water flow in the zero and low density treatments, and in smaller patches, are consistent with this notion.

Herbivory is unlikely to have affected the survivorship of the transplanted juveniles, and while mesograzers such as the small gastropod *Phasianotrochus* spp. were observed on the reefs (but not at high abundances) we did not observe any signs of grazing. Moreover, *Olisthops cyanomelas*–the primary fish species to consume *E*. *radiata* in southern Australia [71]–rarely occurs in south eastern Tasmania and was not observed, while only one small urchin (*Heliocidaris erythrogramma*) was recorded during the study.

In summary, it seems that survival of the transplanted macroscopic juvenile sporophytes was driven by sub-optimal light conditions, and potentially amplified by other deleterious abiotic factors. So, whilst the specific causal mechanisms remains to be tested, degradation of adult *E*. *radiata* habitats appears likely to negatively impact survival of macroscopic juvenile conspecifics. These findings have clear implications for kelp forest demography and resilience, and also contribute to understanding of disturbance-recovery dynamics and kelp forest restoration (i.e. it is not advisable to transplant juvenile *E*. *radiata* sporophytes in the absence of adult conspecifics).

#### Growth of transplanted juvenile sporophytes

**Fig 4:**
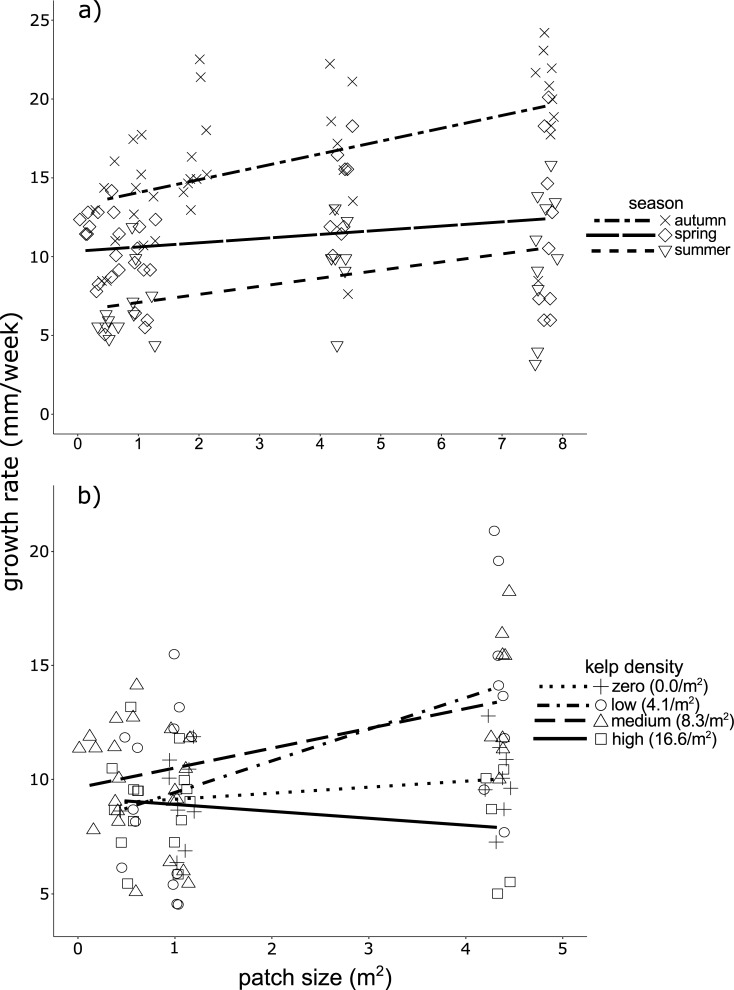
Growth rates of macroscopic juvenile *Ecklonia radiata* sporophytes. Measurements taken 90 days after transplanting to (a) patches at normal kelp density (8.3 kelp/m^2^) across three seasons, and (b) patches of varying kelp density during spring. Note the different y-axis scale.

Growth rates of juvenile sporophytes transplanted into patches of medium density increased with patch size and differed across seasons (Table 2, Fig 4). Juveniles transplanted during autumn had significantly higher growth rates than those transplanted during spring or summer (Table 2). From the juveniles transplanted into the largest patches, since they showed the highest growth rates, mean seasonal growth rates were 19.4 ± 1.4 mm/week (± SE, *n* = 10) in autumn, 11.4 ± 1.8 mm/week (± SE, *n* = 9) in spring and 10.1 ± 1.3 mm/week (± SE, *n* = 10) in summer.

During spring and when tested across all four kelp density treatments, growth rates increased significantly with patch size, but not kelp density (Table 2). However, this effect was evident only for juveniles transplanted into patches of low and medium kelp density (Fig 4) as regression slope analysis indicated that increases in patch size provided no growth benefits for juveniles transplanted to patches of zero (*F*_(1,15)_ = 1.056, *P* = 0.321) or high kelp density (*F*_(1,19)_ = 1.028, *P* = 0.323) (Fig 4B). For the latter, any benefits derived from increases in adult patch size (due to increased engineering) may be overshadowed by concurrent reductions in sub-canopy irradiance (Fig 2A), and thus photosynthesis. While for the former, lack of positive patch size effects is not unexpected (since no adult kelp were present), and is consistent with the notion that increased facilitation by adult kelp with patch size contributed to increased growth rates of transplanted juveniles.

To this end, juveniles transplanted to patches of low kelp density appeared to receive the greatest benefits from increases to patch size (Fig 4). Relative to medium kelp density, patches of low density kelp exhibit elevated levels of irradiance, water flow and sedimentation (Fig 2)–all of which, above a certain threshold, are potential stressors to developing sporophytes [20, 25, 56]. Accordingly, juvenile kelp in these sparser kelp environments are likely to benefit most from increases in patch size, where increased abiotic engineering from adult conspecifics acts to ameliorate potential stressors [21, 72]. These sparse canopies may also facilitate higher growth rates of juveniles relative to denser kelp canopies (Fig 4, [35, 42, 56]), although this may come at a trade-off via reduced survivorship (Fig 3).

Increased growth of juvenile *E*. *radiata* after reductions in adult density may enable swift replenishment of the kelp canopy and its engineering capacity following perturbations. However, this depends on either prompt recruitment of *E*. *radiata* propagules into areas of sparse kelp (which seems unlikely, as discussed below), or presence of a ‘seed bank’ of juvenile sporophytes already primed within the sub-canopy and ready to exploit the newly-formed canopy opening [40, 73].

#### Recruitment and survivorship of microscopic sporophytes

**Fig 5:**
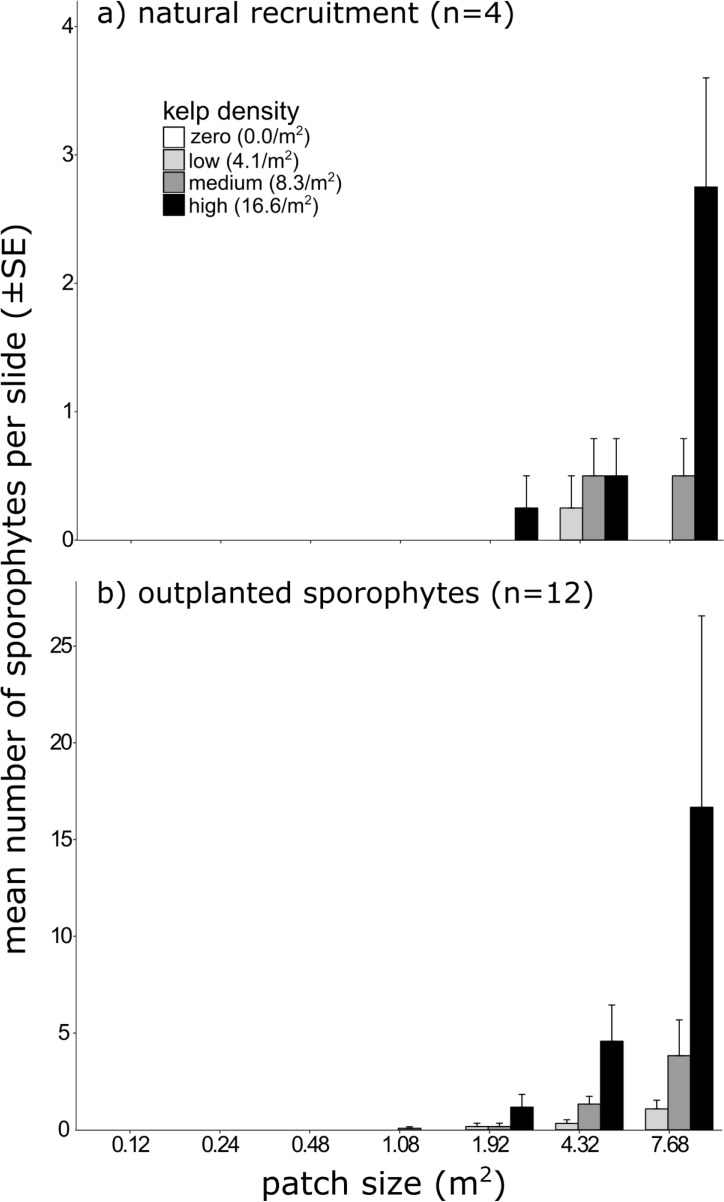
Recruitment and survivorship of microscopic sporophytes on experimental patch reefs. Mean number (± SE) of (a) naturally recruited microscopic *Ecklonia radiata* sporophytes, dependent on patch size and kelp density; and of (b) surviving microscopic sporophytes grown on slides 42 days after outplanting, dependent on patch size and kelp density. Note the different y-axis scales and, that recruitment at patch size 1.08 m^2^ (Fig b) was in the medium density treatment.

After the 42-day outplanting period, only 19 naturally recruited microscopic sporophytes were observed on the control slides. Recruitment was greatest in the largest patch supporting the highest kelp density, while none occurred on patches <1.92 m^2^ (regardless of kelp density) (Fig 5A). No recruitment of microscopic sporophytes occurred on any experimental reefs without adult kelp (Fig 5A); supporting our assumption that reefs were reproductively isolated and that recruitment could be attributed to only the adult kelp on any given reef.

There was a significant interaction between patch size and kelp density (ANCOVA; *F*_(1,24)_ = 9.523, *P* = 0.005), likely reflecting the absence of recruitment on the zero density treatment. Indeed, after omitting the zero density plots the significant interaction was removed, and recruitment increased significantly with patch size, but not kelp density (Table 2). Nonetheless, there were strong kelp density trends, and recruitment occurred mostly on patches of high kelp density (with the largest patch from the treatment accounting for more than half of all observed recruits) (Fig 5A). Higher densities of adult kelp likely influenced supply-side dynamics by producing greater concentration of zoospores and increasing settlement and recruitment success [36, 47, 72]. However, given the significant and stronger effect of patch size (Table 2), it seems that ecosystem engineering and suitability of the abiotic environment was more important for settlement, recruitment and development of the microscopic sporophytes. This precedence of sub-canopy conditions over spore-supply in shaping *E*. *radiata* recruitment has also been observed in previous work [63].

Sedimentation in particular has strong negative effects on the microscopic life stages of kelp, and even small amounts of sediment (e.g. <50 mg/cm^2^) are capable of impairing zoospore attachment and reducing survival and growth of kelp gametophytes [68, 74]. Reduced sediment particle size (<599 μm, [74]) and increased water flow [68] also exacerbate these negative effects, potentially due to greater particle instability and physical damage from abrasion. We certainly observed that the lowest levels of natural recruitment occurred on reefs with the most accumulated sediments and highest sub-canopy flows (Figs 2 and 5A). Related to sedimentation, is the formation of turf algae that trap sediment particles to form a turf-sediment matrix and inhibit recruitment of juvenile *E*. *radiata* [57]. Recent work suggests that differences in chemical conditions at the benthos between turf algae and kelp-dominated assemblages may contribute to this recruitment inhibition [Layton et al. in review].

While light and kelp scour can influence formation of turf algae, they can also have direct effects on kelp settlement and recruitment. Indeed, deleterious effects of elevated irradiance on the microscopic life stages of kelp have been reported [75, 76]. And while scour may damage juvenile kelp via abrasion [28], it can also promote settlement and recruitment by excluding benthic herbivores from beneath the kelp canopy [77, 78]. Overall, we observed that recruitment (and survival) of microscopic sporophytes occurred overwhelmingly on microscope slides that had minimal cover of turf and sediment–hence we suggest that scour was likely a positive force for *E*. *radiata* recruitment on our reefs. Further supporting this assumption is that rates of scour can be minimal in small patches (<3 m^2^) of *E*. *radiata* [Layton et al. in prep.].

Elevated sub-canopy water flow also may directly influence recruitment dynamics of *E*. *radiata* propagules. For example, reduced sub-canopy flow–such as observed on the largest reefs and/or with the densest kelp (Fig 2A)–can facilitate the settlement and survival of propagules of kelp and other macroalgae [68, 79]. Moreover, hydrodynamics beneath intact kelp canopies are characterised by low turbulent mixing and high particle retention relative external conditions [25], which may too facilitate the retention, settlement and recruitment of propagules within the sub-canopy [80–82]. Our observations of decreasing sediment deposition in smaller patches supports this, because the same sub-canopy hydrodynamics that influence the settling of sediment particles likely extends to other small particles such as kelp propagules [25, 60, 79].

By outplanting the already-established lab-cultured microscopic sporophytes, we were able to examine post-settlement survivorship in isolation from effects (e.g. sedimentation, [74]) that may impair propagule attachment and settlement. Nonetheless, survivorship of outplanted microscopic sporophytes was still very low, with only 353 sporophytes surviving from the ~2,500,000 that were outplanted. While high mortality is not unexpected given the ‘low cost-high volume’ reproductive strategy of kelp [40, 83]; these results suggest that impediments to propagule attachment are not the only factors shaping survival of microscopic life stages.

The largest patch supporting the highest kelp density had the most surviving sporophytes, where 200 individuals remained after outplanting (Fig 5B). No microscopic sporophytes survived on any patches <1 m^2^, regardless of kelp density, nor on any patches without adult kelp–further illustrating the importance of adult *E*. *radiata* to development of juvenile conspecifics. Again, a significant interaction between patch size and kelp density (ANCOVA; *F*_(1,24)_ = 9.739, *P* = 0.005) disappeared following omission of the zero density treatment, and survivorship of the outplanted microscopic sporophyte was found to increase significantly with patch size, but not kelp density (Table 2).

Intriguingly, some experimental patches that had surviving outplanted microscopic sporophytes experienced no natural recruitment (Fig 5); indicating that while some patches were suitable for survival of microscopic sporophytes they were either not suitable for settlement/recruitment or failed to receive adequate supply of propagules.

We conclude that recruitment and survivorship of microscopic *E*. *radiata* sporophytes will be negatively affected by reductions to patch size; likely influenced by altered abiotic conditions that influence retention, settlement, attachment and survivorship within the sub-canopy. To this end, provision of suitable habitat and amelioration of physical stress via engineering by adult *E*. *radiata* appears central to facilitating microscopic juvenile conspecifics.

#### Recruitment of macroscopic juvenile sporophytes

**Fig 6:**
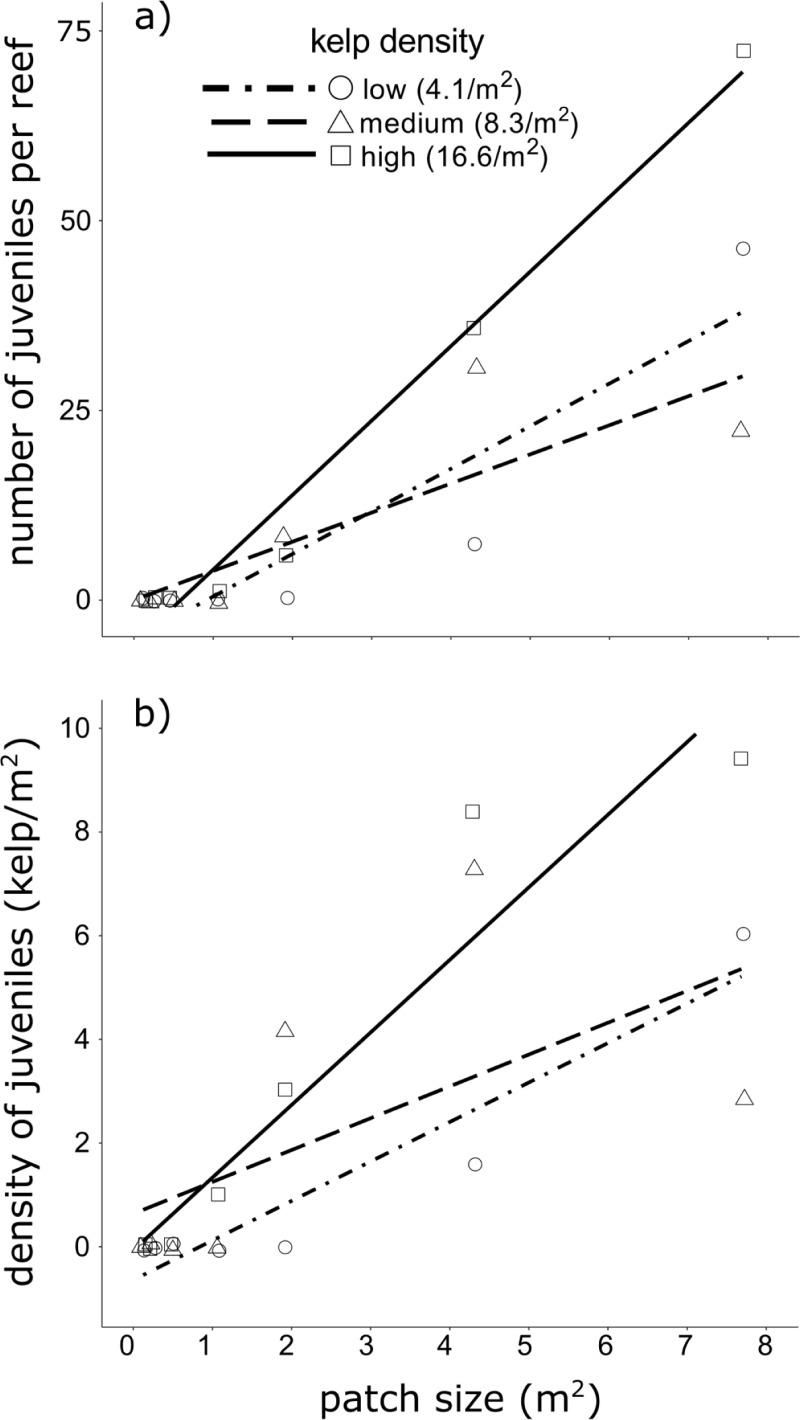
Recruitment of macroscopic juvenile *Ecklonia radiata* sporophytes on different sized patch reefs at end of study. Expressed as (a) absolute number of juveniles per patch and (b) density of recruits (no. individuals/m^2^). Tests were conducted using only density of recruits.

Macroscopic recruits first appeared in early spring, ~7 months after initial transplanting of adult kelp, and originally only on the largest patch supporting the highest density of adult kelp. It was another 2 months until macroscopic recruits were observed on any other patch. No naturally recruited macroscopic sporophytes were observed on any reefs without adult kelp (further reinforcing our assumption of reproductive isolation between reefs) and only a single recruit was ever observed on reefs <1 m^2^. On those reefs where recruitment did occur, the density of recruits was similar to that typically observed in natural *E*. *radiata* habitats [35, 69]. Census of the naturally recruited juveniles at the end of the study illustrated that density of recruits increased significantly with patch size but not kelp density (Table 2, Fig 6). This pattern was mirrored in the earlier censuses, however during that time there was lower recruitment overall and none on any patches of low kelp density. While non-significant, there was a strong trend for patches of high kelp density to have a greater absolute number of recruits and at higher densities, with the greatest number of recruits occurring on the largest reef supporting the highest density of adult kelp during each census (Fig I in S1 File).

Recruitment on patches <2 m^2^ was very rare, indicating there may be a threshold patch size for natural recruitment. Notably, this threshold patch size seems to be smaller at higher densities of adult kelp (Fig 6). A minimum critical patch size for effective recruitment of *E*. *radiata* may arise because of density-dependent processes [47], and reflect ecosystem engineering and local environmental conditions [21].

Overall, the pattern of recruitment of macroscopic sporophytes was similar to that of recruitment and survivorship of the microscopic sporophytes. This was especially so on reefs supporting adult kelp at high density, which showed the highest measures of these demographic processes. The sub-canopy environment on those patches, particularly >2 m^2^, must represent optimal conditions for the recruitment of microscopic and macroscopic juvenile *E*. *radiata*. Interestingly however, our results suggest these conditions are not ideal for the growth of larger macroscopic juvenile sporophytes (Fig 4). In these circumstances, once juvenile *E*. *radiata* reach a certain size their growth must slow or stop to await improved conditions–and thus creating a ‘seed bank’ of juveniles primed within the sub-canopy [35, 40, 69]. Ultimately, recruitment was absent on the experimental reefs lacking sufficient adult kelp, so it is expected that recruitment of *E*. *radiata* into degraded habitats will be significantly compromised by concordant changes to the abiotic and biotic conditions in these environments.

### Environment-engineer feedback

Ecosystem engineering by adult *E*. *radiata* seems to create conditions that facilitate juvenile conspecifics. However, habitat degradation leads to changes in local abiotic and biotic conditions within the sub-canopy that impair demographic rates. Subsequently, reestablishment of an intact kelp canopy is hindered, leading to an increasingly altered sub-canopy environment and a downward spiral of demographic rates. This process represents the loss of a positive environment-engineer feedback, and explains the pattern of impaired recruitment and development of juvenile kelp that we observed to occur with habitat degradation.

Previous studies have highlighted the poor re-establishment of *E*. *radiata* that occurs after large areas (39–314 m^2^) have been cleared of adult canopy [33, 35]. In fact, first signs of recovery can take >9 months to manifest [35], and full canopy recovery >3 years [33]. Notably, those studies describe rapid and opportunistic establishment of other macroalgae into the cleared areas, including species of *Ulva*, *Enteromorpha*, and *Sargassum*. Other work demonstrated that even after heavy inoculation of spores, no *E*. *radiata* recruitment occurred in areas cleared of macroalgal canopy [63]. Widespread loss of adult *E*. *radiata* following habitat disturbance and degradation may therefore result in long-term localised absences of kelp, which may require interspecific facilitation, successional processes, or habitat restoration for reestablishment.

Further hinting at the process of intraspecific facilitation by *E*. *radiata* is that when cleared areas do slowly recover, they are recolonised from the outside inwards [34, 84]–suggesting that new growth and thus settlement, post-settlement survival, and/or recruitment of juvenile *E*. *radiata* is occurring preferentially and more successfully in close proximity to adult conspecifics. Along these same lines, when the cleared areas of *E*. *radiata* are small, recruitment and growth of juveniles, and canopy-replenishment is rapid [35, 42]. As demonstrated by our results however, this rapid replenishment appears dependent on existing juveniles within the sub-canopy at the time of disturbance, rather than opportune recruitment into disturbed areas (see also [69]).

These patterns contrast with some previous observations of the kelp *Macrocystis pyrifera* and *Pterygophora californica*, where cleared patches recolonise from the inside outwards [80, 85], and where juveniles typically do not survive or grow beneath the adult canopy [28, 80, 85]. However, those previous works occurred at deeper depths (>12 m) than examined here, where canopy-juvenile interactions might be more influenced by access to limiting resources (e.g. light for photosynthesis) rather than amelioration of potential stressors. Indeed in shallower environments, juvenile *M*. *pyrifera* may in fact recruit and grow preferentially beneath the adult canopy [86]. Moreover, whilst study of *E*. *radiata* patch dynamics on deeper reefs (~14 m) support the findings presented here, the positive effects of adult patch size on juvenile *E*. *radiata* were more subtle and required longer to manifest–potentially due to more complex hydrodynamics, lower levels of ambient light, and slower formation of turf-forming algae [Layton et al. in prep.]. It follows that along a depth gradient from shallow to deep, population dynamics of kelp may transition from dependency on facilitation to dependency on access to sufficient resources for growth. Thus, experiments replicated across depth and examining juvenile-canopy interactions might reveal details of a ‘facilitation gradient’ in kelp dynamics.

Patch size effects appeared to be of greater significance than kelp density effects in determining the engineering capacity of *E*. *radiata* (Table 2). In fact, our results suggest that demographic processes of juvenile *E*. *radiata* may actually benefit from variations in adult kelp density. In general, patches supporting adult kelp at the mean local (i.e. medium) density provided the ideal environment for juvenile *E*. *radiata*. Yet, patches of sparser kelp appeared to somewhat favour the growth of already established macroscopic juveniles (Fig 4), whilst areas beneath the densest adult canopies had the highest rates of recruitment and survival of microscopic juveniles (Fig 5). The implication of these patterns is that different densities of adult kelp may facilitate separate demographic processes; similar to how variations in canopy density facilitate community richness [20, 42]. Accordingly, mosaics of canopy density might facilitate ontogenetic shifts in habitat requirements and improve overall habitat resilience [40, 73] .

We suggest that survival of the early life stages of *E*. *radiata* may be as much to do with the amelioration of physical stressors as access to abiotic resources. Such that intraspecific facilitation by adult *E*. *radiata* expands the ‘realised niche’ of juvenile conspecifics, through the amelioration of potential stressors such as high sedimentation and deleteriously high light levels [16, 87, 88]. The amelioration of physical stressors and the Stress Gradient Hypothesis have been previously applied to explain facilitation in classically stressful environments, such as the intertidal [14, 16]. Despite the somewhat more benign conditions on subtidal reefs, even a subtle improvement of sub-canopy conditions for juvenile *E*. *radiata* by adult conspecifics could have large implications when applied across the scale of populations [14, 21, 72, 87].

### Conclusion

Ecosystem engineering by canopies of *E*. *radiata* modifies the physical environment to reduce sub-canopy water flow, sedimentation and irradiance. However, the capacity of this kelp to engineer abiotic change is largely dependent on patch size and adult kelp density. We observed that recruitment, growth and survivorship of microscopic and macroscopic juvenile *E*. *radiata* were all impaired by reductions in patch size and, to a lesser extent, kelp density. Even after provision of established propagules and juvenile sporophytes, many of these demographic processes collapsed in the absence of sufficient adult kelp.

The specific mechanisms responsible for the inhibition of the demographic processes of juvenile *E*. *radiata* remains to be explored. However, we illustrate the potential for complex, cumulative and synergistic interactions between stressors. Whilst it seems microscopic stages of *E*. *radiata* are likely to be most affected by loss of adult conspecifics, macroscopic juveniles may possess some capacity (via increased growth rates) to aid rapid recovery of the canopy following a disturbance. However, because this mechanism relies on a ‘seed bank’ of juveniles within the sub-canopy at the time of disturbance, disruption of the microscopic life stages seems likely to impair replenishment of the seed bank and long-term habitat stability.

Our results are consistent with the hypothesis that ecosystem engineering by adult *E*. *radiata* facilitates the development of the juvenile conspecifics. Habitat degradation can impair the ability of *E*. *radiata* to engineer change, causing a breakdown in the positive environment-engineer feedback, leading to the collapse of demographic functions and reduced ecosystem stability and resilience.

## Supporting information

S1 File Results of preliminary analyses and abiotic analyses by season. Table A. Results of preliminary analyses.
Table B. The experimental subsets into which macroscopic juvenile sporophytes were transplanted each season (N = total number of reefs). Winter sampling was attempted, but storms prevented the collection of sufficient data.
Table C. Results of ANCOVAs illustrating no effects of kelp density, patch size, or time of day season on above canopy (i.e. ambient) abiotic factors. Output is or unsaturated models after confirming homogeneity of slopes. Response variable (*Y*) and associated transformation is noted in the first column.
Table D. Results of ANCOVAs testing effects of kelp density, patch size on abiotic factors below the *Ecklonia radiata* canopy by season. Output is for either saturated models (where homogeneity of slopes was rejected) or unsaturated models after confirming homogeneity of slopes. Response variable (*Y*) and associated transformation is noted in the first column. Significant P-values are denoted in bold and with *.
Figure E. Effects of *Ecklonia radiata* patch size and kelp density on sub-canopy water flow across seasons. Sub-canopy flow measured as mean dissolution of plaster clod-cards relative to the paired above-canopy clod-card. Thus 100% represents equal rates of clod-card dissolution below and above the kelp canopy, and values higher or lower than 100% indicate greater or less dissolution below the canopy, respectively. Note the different y-axis scales.
Figure F. Reduction in relative sub-canopy irradiance with increasing density of adult *Ecklonia radiata* shown across all patch sizes. A linear model describing the reduction of light with kelp density is fit to patches ≤1.08 m^2^, while non-linear decay curves are fit to patches ≥ 1.92m^2^. Light is expressed as a percentage of irradiance measured above the canopy in the same patch.
Figure G. Effects of *Ecklonia radiata* patch size and kelp density on sub-canopy sediment deposition across seasons. Data are expressed as percentage of sediment deposition relative to the deposition recorded in a paired sediment trap above the canopy. Thus 100% represents equal rates of deposition below and above the kelp canopy, and values higher or lower than 100% indicate greater or less deposition below the canopy, respectively. Note the different y-axis scales.
Figure H. Effects of *Ecklonia radiata* patch size and kelp density on the depth (mm) of accumulated sediments the reef substratum across seasons.
Figure I. Recruitment of macroscopic juvenile *Ecklonia radiata* sporophytes on different sized patch reefs at half (end of study only), natural and double kelp density, censused during spring, winter and at the end of the study (November 2016). Expressed as absolute number of juveniles per patch (a,b,c) and density of recruits (no. individuals/m^2^) (d,e,f). Note the different y-axis scales.
(DOCX)
