# Supplementary material for: Resilience and stability of kelp forests: The importance of patch dynamics and environment-engineer feedbacks
Source: PLoS One. 2019 Jan 25;14(1):e0210220. doi: 10.1371/journal.pone.0210220 (PMC6347235; doi:10.1371/journal.pone.0210220)
Supplement: S1 File — Table A. Results of preliminary analyses. Table B. The experimental subsets into which macroscopic juvenile sporophytes were transplanted each season (N = total number of reefs). Winter sampling was attempted, but storms prevented the collection of sufficient data. Table C. Results of ANCOVAs illustrating no effects of kelp density, patch size, or time of day season on above canopy (i.e. ambient) abiotic factors. Output is or unsaturated models after confirming homogeneity of slopes. Response variable (Y) and associated transformation is noted in the first column. Table D. Results of ANCOVAs testing effects of kelp density, patch size on abiotic factors below the Ecklonia radiata canopy by season. Output is for either saturated models (where homogeneity of slopes was rejected) or unsaturated models after confirming homogeneity of slopes. Response variable (Y) and associated transformation is noted in the first column. Significant P-values are denoted in bold and with *. Figure E. Effects of Ecklonia radiata patch size and kelp density on sub-canopy water flow across seasons. Sub-canopy flow measured as mean dissolution of plaster clod-cards relative to the paired above-canopy clod-card. Thus 100% represents equal rates of clod-card dissolution below and above the kelp canopy, and values higher or lower than 100% indicate greater or less dissolution below the canopy, respectively. Note the different y-axis scales. Figure F. Reduction in relative sub-canopy irradiance with increasing density of adult Ecklonia radiata shown across all patch sizes. A linear model describing the reduction of light with kelp density is fit to patches ≤1.08 m2, while non-linear decay curves are fit to patches ≥ 1.92m2. Light is expressed as a percentage of irradiance measured above the canopy in the same patch. Figure G. Effects of Ecklonia radiata patch size and kelp density on sub-canopy sediment deposition across seasons. Data are expressed as percentage of sediment deposition relative [file pone.0210220.s001.docx]

**Table A**

| *data analysed* | *F (df)* | *P* |
| --- | --- | --- |
| Clod card initial mass vs mass lost | 2.65 (1,110) | 0.106 |
| Sub-canopy irradiance vs time of day | 1.60 (1,110) | 0.246 |
| **Growth rate of juvenile sporophytes: initial size vs growth rate** | | |
| autumn | 0.979 (1,63) | 0.326 |
| spring | 0.008 (1,99) | 0.929 |
| summer | 1.575 (1,50) | 0.215 |

**Table B**

| *season* | *kelp density (kelp/m^2^)* | *patch size (m^2^)* | *N* |
| --- | --- | --- | --- |
| autumn | medium (8.3) | all patch sizes | 7 |
| spring | zero (0) | 0.48, 1.08, 4.32 | 3 |
|  | low (4.1) | 0.48, 1.08, 4.32 | 3 |
|  | medium (8.3) | 0.12, 0.48, 1.08, 4.32, 7.68 | 5 |
|  | high (16.6) | 0.48, 1.08, 4.32 | 3 |
| summer | zero (0) | 0.48, 1.08, 4.32 | 3 |
|  | low (4.1) | 0.48, 1.08, 4.32 | 3 |
|  | medium (8.3) | 0.12, 0.48, 1.08, 4.32, 7.68 | 5 |
|  | high (16.6) | 0.48, 1.08, 4.32 | 3 |

**Table C**

| *data analysed* | *factor* | *SS (df)* | *F* | *P* |
| --- | --- | --- | --- | --- |
| **WATER FLOW** | | | | |
| winter  (*Y*)^2.00^ | kelp density | 86907 (1,25) | 1.240 | 0.276 |
|  | patch size | 190274 (1,25) | 2.715 | 0.112 |
| spring  (*Y*)^1.80^ | kelp density | 911 (1,25) | 0.149 | 0.703 |
|  | patch size | 22882 (1,25) | 3.739 | 0.065 |
| **SEDIMENT DEPOSITION** | | | | |
| autumn  (*Y*)^-0.13^ | kelp density | 0.001 (1,24) | 0.536 | 0.471 |
|  | patch size | 0.001 (1,24) | 1.140 | 0.296 |
| winter | kelp density | 933 (1,22) | 0.257 | 0.618 |
|  | patch size | 13042 (1,22) | 3.587 | 0.071 |
| spring  (*Y*)^0.45^ | kelp density | 4.400 (1,24) | 2.675 | 0.071 |
|  | patch size | 0.001 (1,24) | 0.005 | 0.945 |
| summer  log(*Y*) | kelp density | 0.031 (1,25) | 0.244 | 0.625 |
|  | patch size | 0.001 (1,25) | 0.001 | 0.924 |
| **IRRADIANCE** | | | | |
| spring  (*Y*)^-0.65^ | kelp density | 1.51^e-5^ (1,109) | 0.730 | 0.395 |
|  | patch size | 7.24^e-5^ (1,109) | 3.503 | 0.064 |
| spring  (*Y*)^-0.65^ | time of day | 4.40e^-6^ (1,109) | 0.212 | 0.672 |
|  | patch size | 4.65e^-5^ (1,109) | 3.683 | 0.058 |

**Table D**

| *data analysed* | *factor* | *SS (df)* | *F* | *P* |
| --- | --- | --- | --- | --- |
| **WATER FLOW** | | | | |
| winter (double density omitted)  (*Y*)^5.25^ | kelp density | 4.70e^19^ (1,18) | 2.825 | 0.110 |
|  | log_2_(patch size) | 8.58e^20^ (1,18) | 51.589 | **<0.001*** |
| spring (double density omitted)  (*Y*)^3.75^ | kelp density | 5.33e^13^ (1,18) | 6.338 | **0.022*** |
|  | patch size | 2.54e^14^ (1,18) | 30.256 | **<0.001*** |
| **SEDIMENT DEPOSITION** | | | | |
| autumn  (*Y*)^0.65^ | kelp density | 26.2 (1,24) | 2.478 | 0.128 |
|  | patch size | 63.9 (1,24) | 6.038 | **0.022*** |
| winter  (*Y*)^-0.15^ | kelp density | 0.01 (1,22) | 2.557 | 0.124 |
|  | patch size | 0.01 (1,22) | 5.281 | **0.031*** |
| spring  (*Y*)^0.60^ | kelp density | 204.22 (1,25) | 17.384 | **<0.001*** |
|  | patch size | 24.67 (1,25) | 2.100 | 0.160 |
| summer  (*Y*)^0.55^ | kelp density | 56.71 (1,25) | 4.304 | **0.048*** |
|  | patch size | 7.09 (1,25) | 0.538 | 0.470 |
| **SEDIMENT ACCUMULATION** | | | | |
| spring (zero density omitted)  (*Y+*0.1)^0.60^ | kelp density | 1.35 (1,102) | 1.480 | 0.227 |
|  | log_2_(patch size) | 8.39 (1,102) | 9.171 | **0.003*** |
| summer  (*Y+*0.1)^0.35^ | kelp density x log_2_(patch size) | 2.41 (1,136) | 11.560 | **<0.001*** |
| **IRRADIANCE** | | | | |
| spring  (*Y*)^0.15^ | kelp density x log_2_(patch size) | 0.89 (1,108) | 71.120 | **<0.001*** |


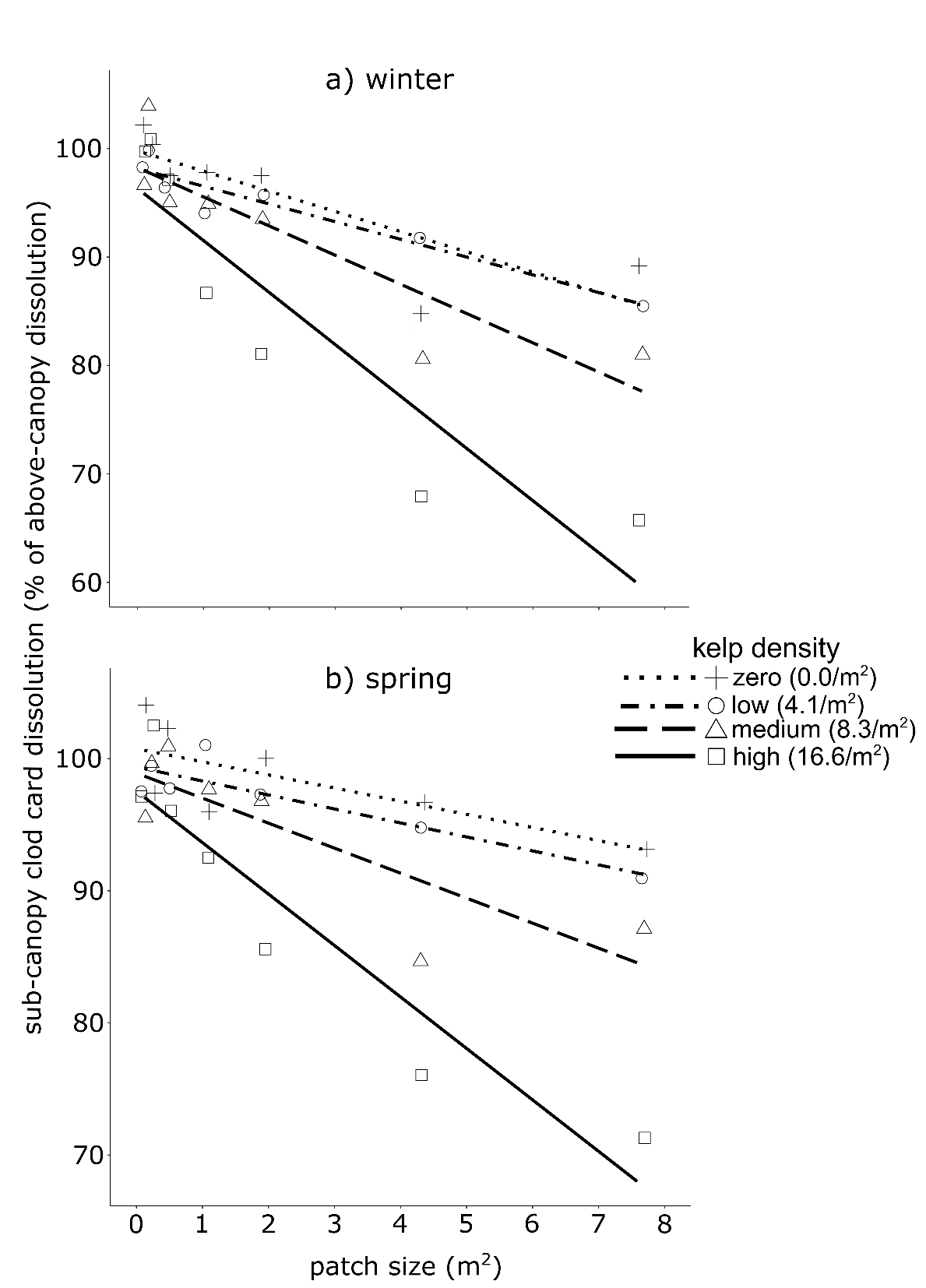


**Figure E**


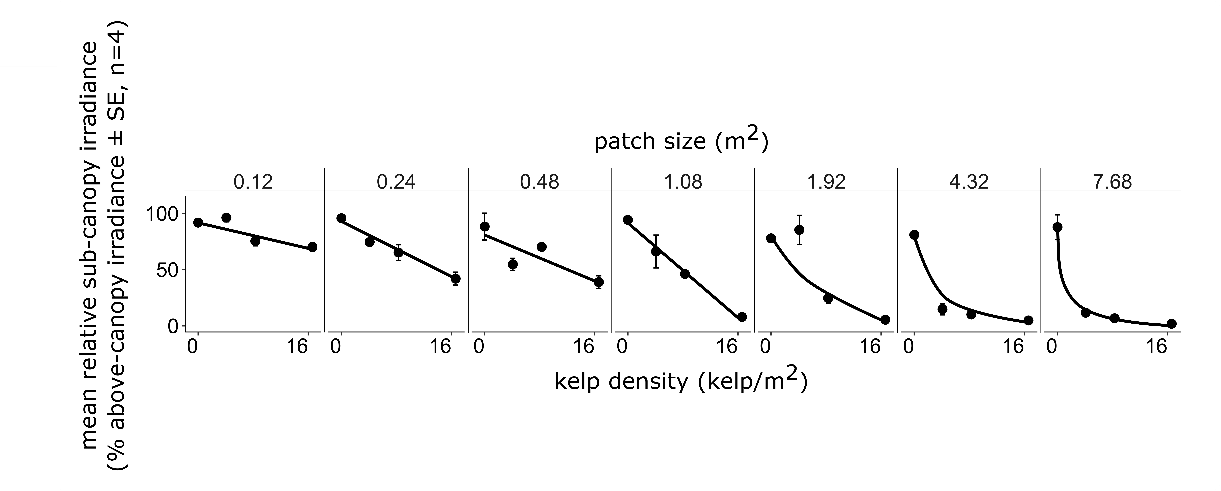


**Figure F**


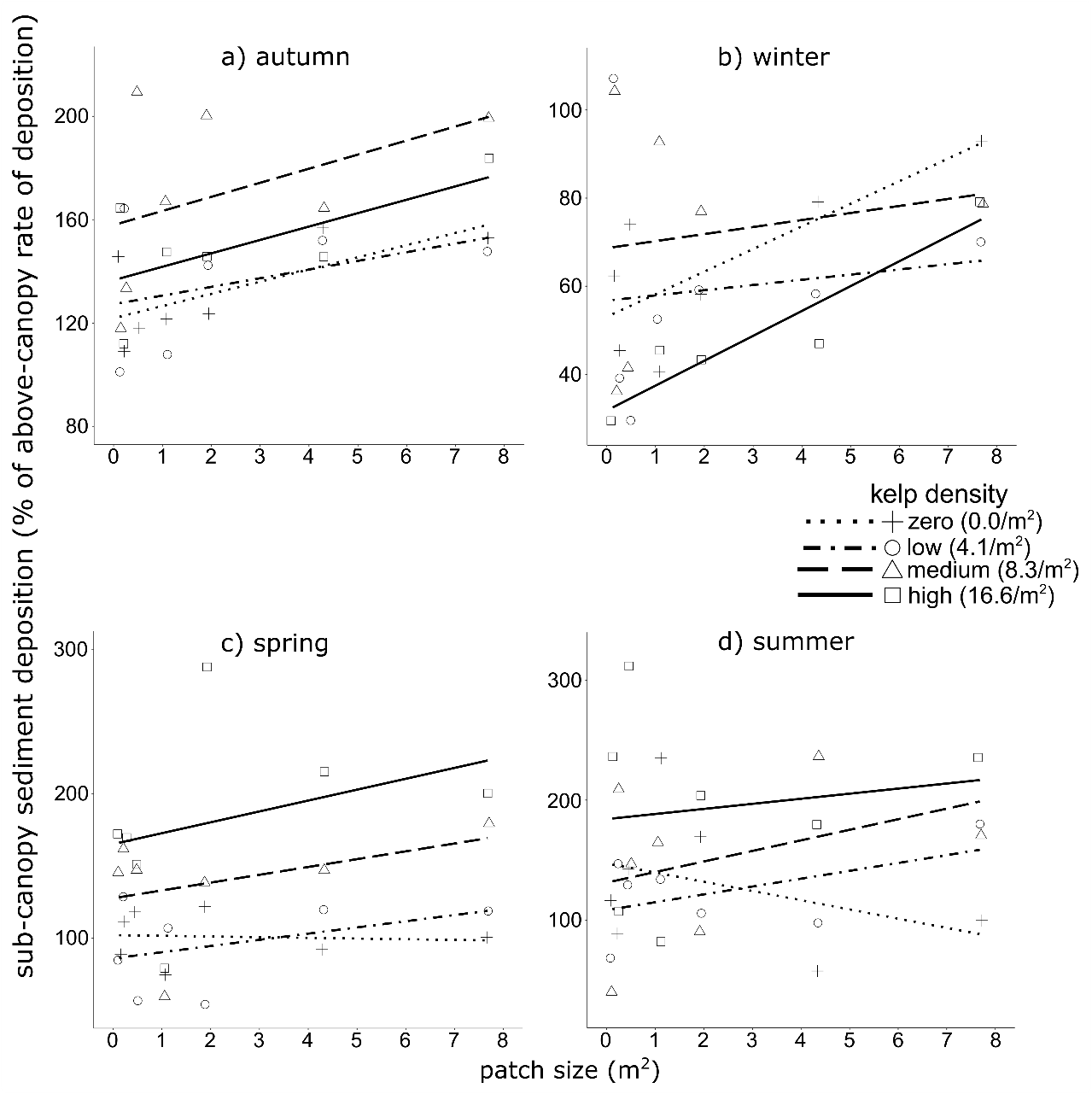


**Figure G**

*
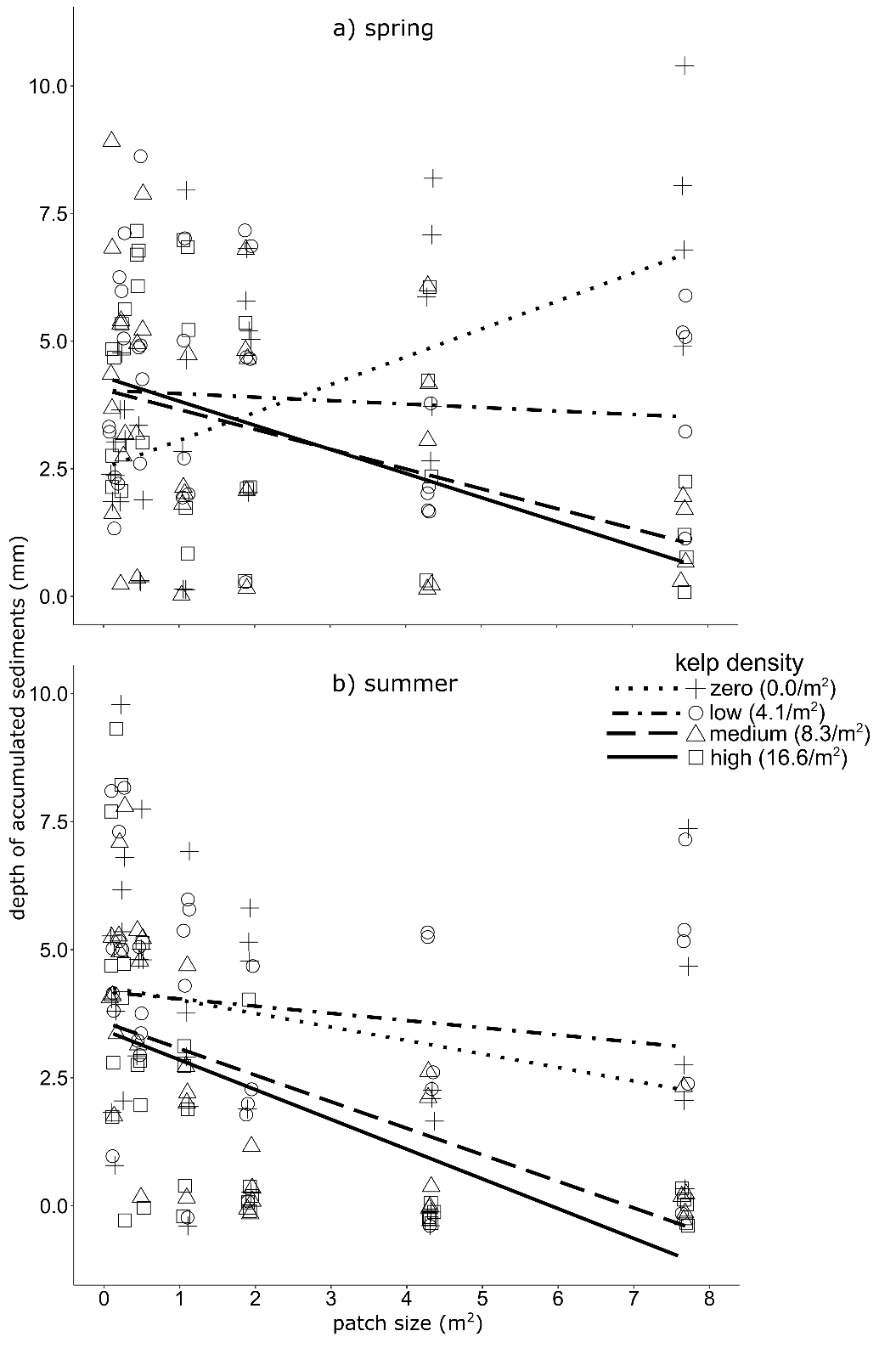
*

**Figure H**


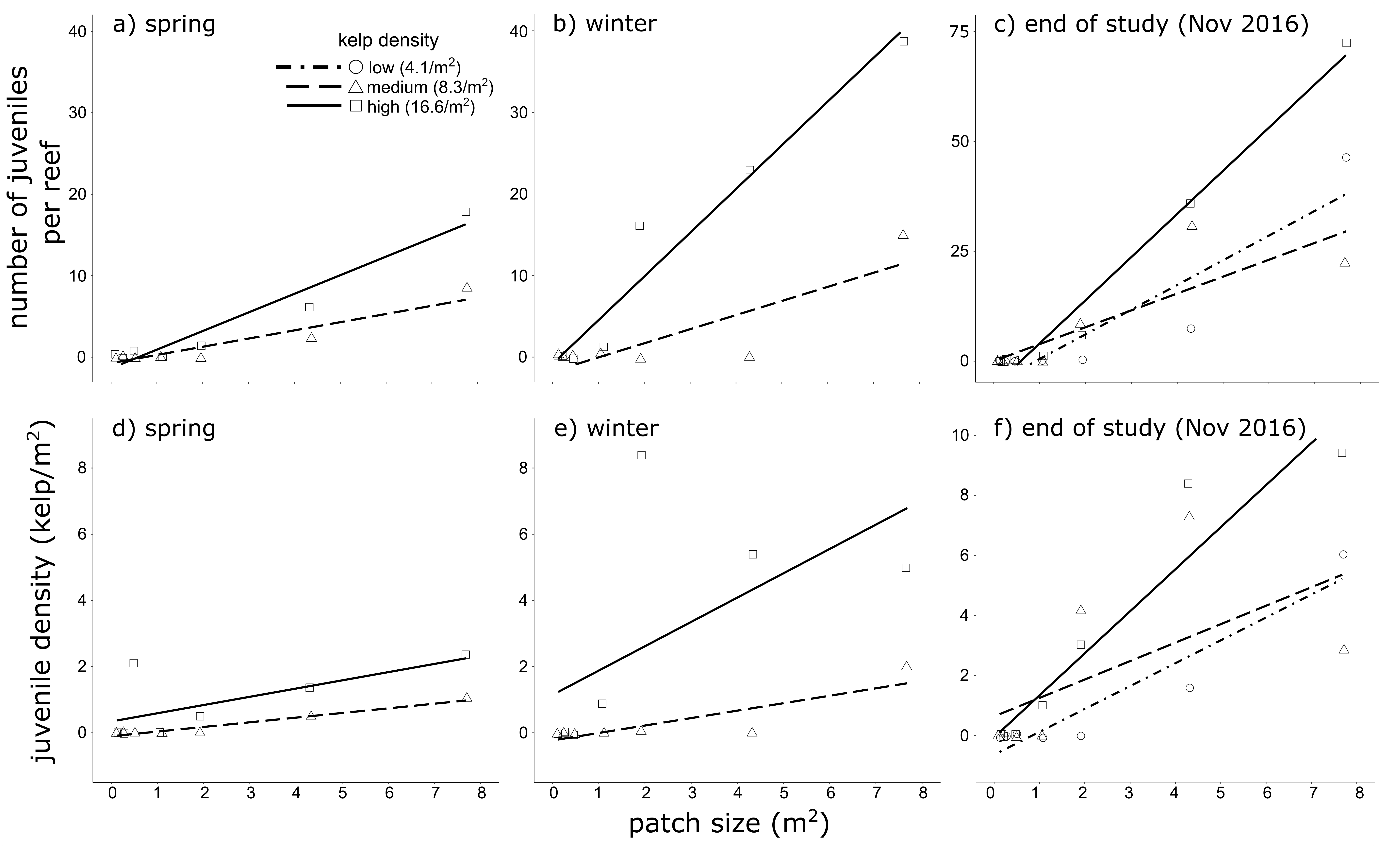


**Figure I**
